# A noise-resisted scheme of dynamical decoupling pulses for quantum memories

**DOI:** 10.1038/s41598-020-72071-x

**Published:** 2020-09-15

**Authors:** Bo Gong, Tao Tu, Xing-Yu Zhu, Ao-lin Guo, Zong-quan Zhou, Guang-Can Guo, Chuan-Feng Li

**Affiliations:** 1 Key Laboratory of Quantum Information, University of Science and Technology of China, Chinese Academy of Sciences, Hefei, 230026 People’s Republic of China; 2 Department of Physics and Astronomy, University of California at Los Angeles, Los Angeles, CA 90095 USA

**Keywords:** Optical techniques, Optical physics, Quantum physics

## Abstract

Stable quantum memories that capable of storing quantum information for long time scales are an essential building block for an array of potential applications. The long memory time are usually achieved via dynamical decoupling technique involving decoupling of the memory states from its local environment. However, because this process is strongly limited by the errors in the pulses, an noise-protected scheme remains challenging in the field of quantum memories. Here we propose a scheme to design a noise-resisted $\pi$ pulse, which features high fidelity exceeding $99.9\%$ under realistic situations. Using this $\pi$ pulse we can generate different dynamical decoupling sequences that preserve high fidelity for long time scales. The versatility, robustness, and potential scalability of this method may allow for various applications in quantum memories technology.

## Introduction

Quantum memories are building blocks for various quantum information processing^1–3^. These devices can be used to store the quantum information for programmable time to synchronize probabilistic processes, which is central for the scalability of distributed quantum computing and long distance quantum communication^4–6^. To characterize the performance of a quantum memory, one can use several figures of merit: efficiency, fidelity, capacity and storage time^1–3^. For example, in a quantum repeater node for large scale quantum networks, one requires a long-lived quantum memory, where the storage time in the node must be longer than the entanglement distribution time between distant nodes. Thus to extend the storage time is a key task in general applications of quantum memories.

There are a variety of platforms to implement quantum memories, among them rare-earth ions in crystals are a promising candidate^7–9^. These ions in solids have strong light-matter interactions to enable high efficiency of the quantum storage and retrieval^10–13^. More importantly, the electron spins or nuclear spins of the ions have long coherence time to serve as a long-lived memory^14, 15^. However, in ensembles the spin states are subject to inhomogeneous spin broadening and fluctuations in the environment, which is the main limitation of the storage time. To address this problem, one can use dynamical decoupling (DD) techniques. A DD pulse sequence, which applies a series of population inversion pulses (e.g. $\pi$ pulses), can protect the system from the dephasing processes due to the surrounding bath^16^. Thus the storage time of the spins can be extended by orders of magnitude. A prominent example is the achievement of coherence time of Eu ions in solids about hours time scales by mitigating the dephasing with DD pulse sequences^17^.

However, the efficiency of the DD scheme strongly depends on the errors in the pulses themselves. Errors in the $\pi$ pulses significantly reduce the fidelity of the DD sequences^18, 19^, which thereby reduces the achieved storage time^15^, and causes an additional source of photon noise^20^. Therefore developing the precise and noise-resisted DD protocols remains an outstanding challenge for quantum memories.

Here we propose a scheme to construct the DD pulse sequence using self-corrected $\pi$ pulse instead of simple form. Firstly, we introduce the basic idea to design a composite pulse that is immune to the imperfections in the pulse, making high fidelity manipulations applicable. Then, we investigate two representative implements of noise-resisted $\pi$ pulses. We provide the extensive simulations of the pulse parameters to achieve high fidelity beyond $99.9\%$ in realistic situations. Based on these results, we insert the $\pi$ pulses into the DD sequences and study the performance of various DD sequence. We show that the noise-protected $\pi$ pulses are at the heart of the DD sequence, thereby provide the required high fidelity for long-time applications. We note that the conventional DD method is a general approach^21–29^ to protect the states of a two-level system in various platforms, such as NV centers^30–32^, semiconductor quantum dots^33^, trapped ions^34^, and superconducting qubits^35^, which can be widely used in manipulation of spins, sensing, and spectroscopy^16, 36^. Thus, the designed scheme can be employed to different types of quantum memories and applications.

## Results

### Model for a quantum memory

**Figure 1:**
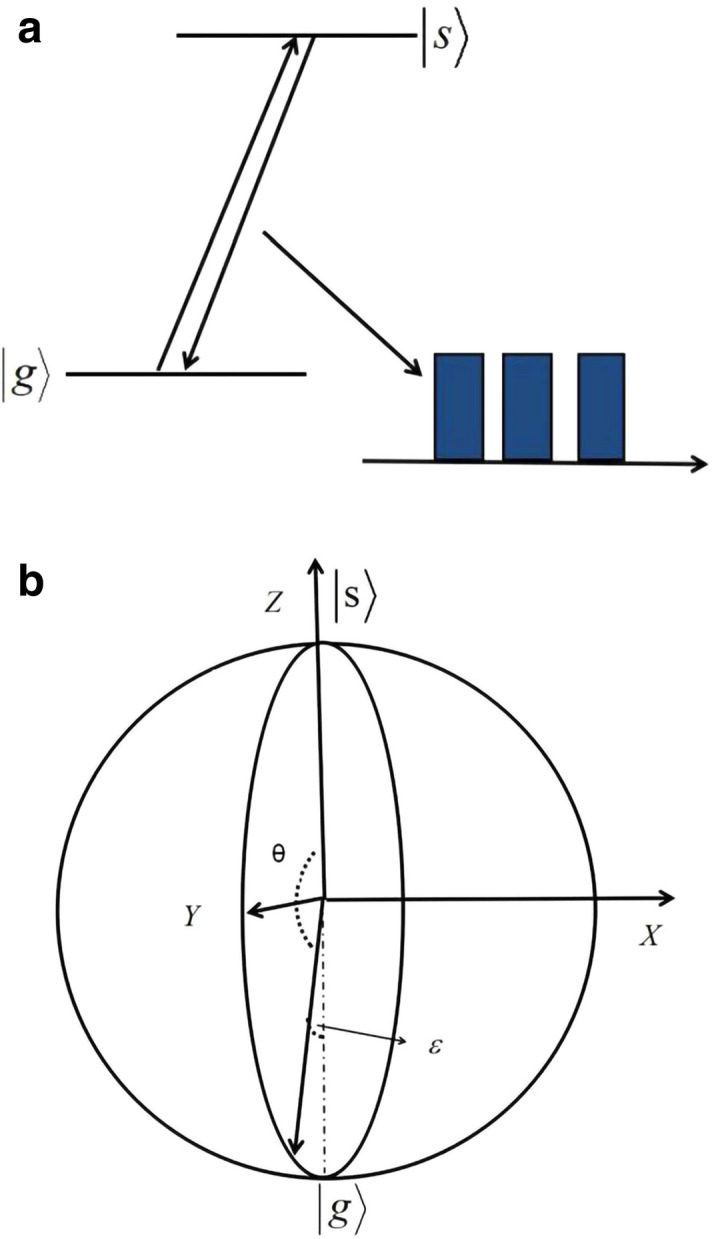
**(a)** The two-level structure for a typical quantum memory, where two spin states $\left| g\right\rangle$ and $\left| s\right\rangle$ are manipulated by a DD pulse sequence to extend the storage time. **(b)** The sketch of a noisy $\pi$ pulse. Here, it is a rotation around *x* axis with an angle $\theta =\pi$. Due to the effects of noises, there are errors in both axes and angle of the rotation. Minimizing this pulse error is an important target for the DD sequence comprised of multiple $\pi$ pulses.

In the present paper, we use rare-earth ions doped crystals, such as $\hbox {Pr}^{3+} :\hbox {Y}_{2}\hbox {SiO}_{5}$ or $\hbox {Eu}^{3+}:\hbox {Y}_{2}\hbox {SiO}_{5}$, as an example of the quantum memory platforms^14, 15^. These systems use an ensemble of ions with a two-level structure involving two spin states $\left| g\right\rangle$ and $\left| s\right\rangle$ as shown in Fig. 1a. We use spin states $\left| g\right\rangle$ and $\left| s\right\rangle$ for long-term storage as these states have long coherence times. However, the spin states in solid systems are usually subject to decoherence processes, which stems from a variety of sources, including charge noise, nuclear spin fluctuations, thermal fluctuations, etc. In this case, a leading source of noise is fluctuations in the spin energy splitting due to interactions with neighboring nuclear spins^17^. To overcome this dephasing, we can apply DD sequences (i.e., a series of microwave or radio-frequency $\pi$ pulses) on these spin states. The $\pi$ pulses swap the populations between the spin states $\left| g\right\rangle$ and $\left| s\right\rangle$. In the rotating frame of the control pulses, the model Hamiltonian for the manipulation of the spin states can be expressed as^15, 16, 20^

1 $$\begin{aligned} H_{0}(t)=\frac{\Delta }{2}\sigma _{z}+\frac{\Omega }{2}\sigma _{x}, \end{aligned}$$

where $\sigma _{x}$ and $\sigma _{z}$ are the Pauli operators defined in the subspace of $\left| g\right\rangle$ and $\left| s\right\rangle$, $\Delta$ is the frequency detuning of the pulse (i.e., the energy splitting between the two levels), and $\Omega$ is the driving amplitude of the pulse (i.e., the Rabi frequency between the two levels). The evolution of such system is determined by the propagator

2 $$\begin{aligned} U_{0}(\Delta {\hat{z}}+\Omega {\hat{x}},\theta )=\exp \left[ -i\left( \frac{ \Delta }{2}\sigma _{z}+\frac{\Omega }{2}\sigma _{x}\right) \frac{\theta }{ \sqrt{\Delta ^{2}+\Omega ^{2}}}\right] , \end{aligned}$$

which produces a rotation by angle $\theta$ around the axis $\Delta {\hat{z}} +\Omega {\hat{x}}$, as shown in Fig. 1b. Here for convenience, we use the subscript 0 to denote the case without noises and set the Planck constant $\hslash =1$.  Typically we assume that the driving amplitude is much larger than the frequency detuning as $\Omega \gg \Delta$, then we can achieve a $\pi$ pulse around the axis ${\hat{x}}$ by holding $\Omega$ constant for a time $\pi /\Omega$.

However, this assumption or approximation is not valid in various quantum memory schemes because of the high fidelity we need. More importantly, the system undergoes two kinds of noises: On one hand, the spin inhomogeneous broadening and surrounding noises add a small fluctuation term $\delta \Delta$ to the Hamiltonian. On the other hand, the imperfections lead to deformation of the control pulses and in turn the energy level structure. In addition to detuning error, these control noises also lead to perturbation $\delta \Omega$ about the amplitude. The fluctuations of the system parameters modify both axes and angle of the rotation, as shown in Fig. 1b. This error significantly reduces the fidelity of $\pi$ pulses and thereby DD pulse sequences. To address this problem, we design a composite pulse to achieve the high fidelity operation since it is noise-resisted.

### General strategy for design of a noise-protected rotation

The purpose is to design a series of composite pulses in such a way that all the error terms are cancelled to each other. There are many approaches to design these composite pulses^18, 19^, while we follow the idea of dynamical corrected gates to outline the detailed procedure for the scheme^37, 38^.

(i) Start with a noisy elementary pulse operator. The Hamiltonian with noises can be written as

3 $$\begin{aligned} H(t)=\frac{(\Delta +\delta \Delta )}{2}\sigma _{z}+\frac{(\Omega +\delta \Omega )}{2}\sigma _{x}, \end{aligned}$$

and a single pulse can be expressed an operator as

4 $$\begin{aligned} U(\Delta {\hat{z}}+\Omega {\hat{x}},\theta )=\exp \left[ -i\left( \frac{\Delta +\delta \Delta }{2}\sigma _{z}+\frac{\Omega +\delta \Omega }{2}\sigma _{x}\right) \frac{ \theta }{\sqrt{\Omega ^{2}+\Delta ^{2}}}\right] . \end{aligned}$$

In practice, the system parameters $\Delta$ and $\Omega$ can be controlled dynamically, whose values are bounded between zero and a certain maximal positive value. This will lead to constraint on the design of the adjustable parameters. In our calculations below, we treat these two noise sources as quasi-static, where each noise is modelled by a random value from a Gaussian distribution with standard deviation. The time evolution is averaged over many repetitions to give the final result; for each repetition new values for the static noise are sampled.

(ii) Determine the error terms of the noisy elementary operator. We can expand the operator to the first order of small fluctuations $\delta \Delta$ and $\delta \Omega$,

5 $$\begin{aligned} U(\Delta {\hat{z}}+\Omega {\hat{x}},\theta )&= {} \exp \left[ -i\left( \frac{\Delta }{2}\sigma _{z}+\frac{\Omega }{2}\sigma _{x}\right) \frac{\theta }{\sqrt{\Omega ^{2}+\Delta ^{2}}}\right] \nonumber \\&\quad \times \left( I_{0}-i\sum _{k=x,y,z}\varepsilon _{k}\sigma _{k}\right) \nonumber \\&= {} U_{0}(\Delta {\hat{z}}+\Omega {\hat{x}},\theta ) \left( I_{0}-i\sum _{k=x,y,z}\varepsilon _{k}\sigma _{k}\right) . \end{aligned}$$

Here $U_{0}(\Delta {\hat{z}}+\Omega {\hat{x}},\theta )$ is the noiseless pulse operator, $I_{0}$ is the ordinary identity operator, $\sigma _{k}$ is the Pauli operator and $\varepsilon _{k}$ is the error term due to the noise sources $\delta \Delta$ and $\delta \Omega$. (We can calculate the error terms in these operators as a function of $\delta \Delta$ and $\delta \Omega$, please see details in the Supplementary Information).

(iii) Use a series of pulses to construct a noisy identity operator. For convenience, we make use of a recursive form of the elementary pulse $U(\Delta {\hat{z}}+\Omega {\hat{x}},\theta )$ to generate an identity operator

6 $$\begin{aligned} {\tilde{I}}^{(n)}&= {} U(\Delta _{n}{\hat{z}}+\Omega _{n}{\hat{x}},m_{n}\pi +\theta _{n}){\tilde{I}}^{(n-1)} \nonumber \\&\quad \times U(\Delta _{n}{\hat{z}}+\Omega _{n}{\hat{x}},m_{n}\pi -\theta _{n})\nonumber \\&= {} U(\Delta _{n}{\hat{z}}+\Omega _{n}{\hat{x}},m_{n}\pi +\theta _{n}) \nonumber \\&\quad \times U(\Delta _{n-1}{\hat{z}}+\Omega _{n-1}{\hat{x}},m_{n-1}\pi +\theta _{n-1})... \nonumber \\&\quad \times U(\Delta _{1}{\hat{z}}+\Omega _{1}{\hat{x}},m_{1}\pi +\theta _{1})U(\Delta _{0}{\hat{z}}+\Omega _{0}{\hat{x}},2m_{0}\pi ) \nonumber \\&\quad \times U(\Delta _{1}{\hat{z}}+\Omega _{1}{\hat{x}},m_{1}\pi -\theta _{1})...\nonumber \\&\quad \times U(\Delta _{n-1}{\hat{z}}+\Omega _{n-1}{\hat{x}},m_{n-1}\pi -\theta _{n-1}) \nonumber \\&\quad \times U(\Delta _{n}{\hat{z}}+\Omega _{n}{\hat{x}},m_{n}\pi -\theta _{n}). \end{aligned}$$

Here *n* denotes the level of the recursive form, $m_{n}$ is an integer, $\Delta _{n}$, $\Omega _{n}$ and $\,\theta _{n}$ are the rotation axis and the rotation angle which are to be determined. We note that this recursive form has several advantages: First, it has an intuitive physical picture which means that the composite pulse consists of a series of rotations with different axes and angles on the Bloch sphere. Secondly, it offers free parameters $\Delta _{n}$, $\Omega _{n}$ and $\theta _{n}$ for each pulse to allow for noise cancellation of the whole pulse sequence.

(iv) Determine the error terms of the noisy identity operator. In general, we can separate the error terms from the identity operator as the following

7 $$\begin{aligned} {\tilde{I}}^{(n)}=I_{0}-i\sum _{k=x,y,z}\Theta _{k}^{(n)}\sigma _{k}, \end{aligned}$$

where $I_{0}$ is the standard identity operator, $\Theta _{k}$ is the error term that comes from the accumulation of the noise terms $\delta \Delta$ and $\delta \Omega$. In the calculations, we can perform a matrix multiplication to obtain $\Theta _{k}$ as a function of the pulse parameters $\Delta _{n}$, $\Omega _{n}$ and $\theta _{n}$.

(v) Adjust the free parameters to realize that the sum of all the error terms from each pulse equals zero. For the target operation $U(\Delta _{t} {\hat{z}}+\Omega _{t}{\hat{x}},\theta _{t})$, we use the entire composite pulse as $U_{c}={\tilde{I}}^{(n)}U(\Delta _{t}{\hat{z}}+\Omega _{t}{\hat{x}},\theta _{t})$ and obtain the expression

8 $$\begin{aligned} U_{c}=U_{0}(\Delta _{t}{\hat{z}}+\Omega _{t}{\hat{x}},\theta _{t}) \left( I_{0}-i\sum _{k=x,y,z}\Gamma _{k}\sigma _{k}\right) . \end{aligned}$$

Here the error term $\Gamma _{k}$ is also a function of the free pulse parameters. To the first order of $\delta \Delta$ and $\delta \Omega$, $\Gamma _{k}=\varepsilon _{k}+\Theta _{k}^{(n)}$, we can choose the parameters to fulfill the equation

9 $$\begin{aligned} \varepsilon _{k}+\Theta _{k}=0. \end{aligned}$$

Therefore the whole pulse $U_{c}$ achieves the target operation $U_{0}(\Delta _{t}{\hat{z}}+\Omega _{t}{\hat{x}},\theta _{t})$ which is also immune to the leading order of noises.

### Two representative cases of a noise-resisted ${\pi }$ pulse

In the following, we apply the above general procedure to explicitly construct a noise-resistant $\pi$ pulse around *x* axis (i.e., the target operation is $U(\Delta _{t}{\hat{z}}+\Omega _{t}{\hat{x}},\theta _{t})$ where $\Delta _{t}=0$, $\Omega _{t}=1$, $\theta _{t}=\pi$). Here we discuss two representative cases which cover a variety of realistic situations. In the first case we set the driving amplitude $\Omega =1$ as constant and the frequency detuning $\Delta$ as the tunable parameter.

We start from the elementary operator and we construct a five-level pulse sequence for an identity operator

10 $$\begin{aligned} {\tilde{I}}^{(5)}&= {} U(\Delta _{t}{\hat{z}}+{\hat{x}},\pi +\frac{\theta _{t}}{2} )U(\Delta _{4}{\hat{z}}+{\hat{x}},\pi )U(\Delta _{3}{\hat{z}}+{\hat{x}},\pi ) \nonumber \\&\quad \times U(\Delta _{2}{\hat{z}}+{\hat{x}},\pi )U(\Delta _{1}{\hat{z}}+{\hat{x}},\pi )U(\Delta _{0}{\hat{z}}+{\hat{x}},4\pi ) \nonumber \\&\quad \times U(\Delta _{1}{\hat{z}}+{\hat{x}},\pi )U(\Delta _{2}{\hat{z}}+{\hat{x}},\pi )U(\Delta _{3}{\hat{z}}+{\hat{x}},\pi ) \nonumber \\&\quad \times U(\Delta _{4}{\hat{z}}+{\hat{x}},\pi )U\left( \Delta _{t}{\hat{z}}+{\hat{x}},\pi - \frac{\theta _{t}}{2}\right) . \end{aligned}$$

Here we choose the angle parameters as $m_{0}=2$, $m_{1,2,3,4,5}=1$, $\theta _{1,2,3,4}=0$, $\theta _{5}=\frac{\theta _{t}}{2}$ and one detuning parameter as $\Delta _{5}=\Delta _{t}$, and use other detuning quantities $\Delta _{0,1,2,3,4}$ as the free parameters.

We note that due to the nonlinearity of the equations of the free parameters, it is not guaranteed that the solutions are real and non-negative as required. For example, if one choose 2 or 3-level pulse sequence, numerically solving these equations always gives non-physical solution of the parameters. Actually, a longer sequence such as 5-level pulse sequence offers sufficient freedom that noise cancellation is always possible for all cases we studied, as explicitly demonstrated in the present work. Therefore, choosing the length of the pulse sequence is a tradeoff between longer pulse for flexibility and shorter pulse for convenience.

Thus we obtain the composite pulse as

11 $$\begin{aligned} U_{cp1}&= {} {\tilde{I}}^{(5)}U(\Delta _{t}{\hat{z}}+{\hat{x}},\theta _{t}) \nonumber \\&= {} U\left( \Delta _{t}{\hat{z}}+{\hat{x}},\pi +\frac{\theta _{t}}{2}\right) U(\Delta _{4}\hat{ z}+{\hat{x}},\pi )U(\Delta _{3}{\hat{z}}+{\hat{x}},\pi ) \nonumber \\&\quad \times U(\Delta _{2}{\hat{z}}+{\hat{x}},\pi )U(\Delta _{1}{\hat{z}}+{\hat{x}},\pi )U(\Delta _{0}{\hat{z}}+{\hat{x}},4\pi ) \nonumber \\&\quad \times U(\Delta _{1}{\hat{z}}+{\hat{x}},\pi )U(\Delta _{2}{\hat{z}}+{\hat{x}},\pi )U(\Delta _{3}{\hat{z}}+{\hat{x}},\pi ) \nonumber \\&\quad \times U(\Delta _{4}{\hat{z}}+{\hat{x}},\pi )U\left( \Delta _{t}{\hat{z}}+{\hat{x}},\pi + \frac{\theta _{t}}{2}\right) , \end{aligned}$$

where the subscript denotes the entire pulse as CP1 for clarity. We note that the whole composite pulse is a symmetric pulse sequence. The noise cancellation requirement $\varepsilon _{k}+\Theta _{k}=0$ leads to a set of nonlinear coupled equations for the free parameters $\Delta _{0,1,2,3,4}$. By numerically solving these equations, we obtain the wanted frequency detunings and show the results in Table 1 (here we set $\Omega =1$ as the basic energy unit).

**Table 1:** Parameters for the designed composite pulse in Eq. (11).

Rotation	$\theta _{t}$	$\Delta _{0}$	$\Delta _{1}$	$\Delta _{2}$	$\Delta _{3}$	$\Delta _{4}$
U(${\hat{x}}$,$\theta _{t}$)	$\pi$	0.5290	7.2860	0	3.0639	0.86059

**Table 2:** Parameters for the designed composite pulse in Eq. (14).

Rotation	$\theta _{t}$	$\Omega _{0}$	$\Omega _{1}$	$\Omega _{2}$	$\Omega _{3}$	$\Omega _{4}$
U(${\hat{x}}$,$\theta _{t}$)	$\pi$	0.6448	3.44043	0	2.46174	0.3122

Now we consider the second case where we set the frequency detuning $\Delta =1$ as constant and the driving amplitude $\Omega$ as the adjustable parameter. Since the detuning $\Delta$ has a nonzero value, we can not directly achieve a rotation around *x* axis regardless of any value of $\Omega$. Alternatively we use a three-step pulse to implement a *x*-axis rotation as

12 $$\begin{aligned} U(\Omega _{t}{\hat{x}},\theta _{t})=U(\Delta _{t}{\hat{z}}+\Omega _{t}{\hat{x}} ,\pi )U(\Delta _{t}{\hat{z}},\theta _{t})U(\Delta _{t}{\hat{z}}+\Omega _{t}{\hat{x}},\pi ), \end{aligned}$$

(i.e., the target operation is $U(\Omega _{t}{\hat{x}},\theta _{t})$ where $\Delta _{t}=1$, $\Omega _{t}=1$, $\theta _{t}=\pi$).

**Figure 2:**
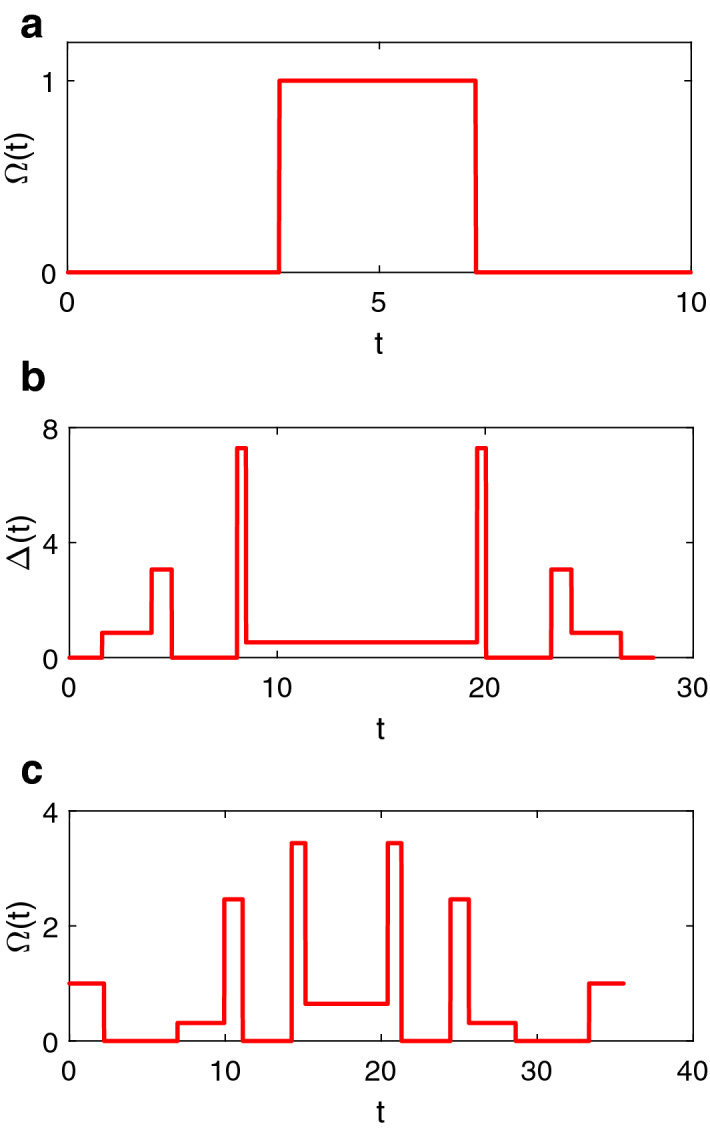
**(a)** The commonly used rectangular $\pi$ pulse. Setting $\Delta =0$ and $\Omega =1$ for a duration of $\pi$ leads to a typical $\pi$ pulse.**(b)** The first type of noise-resisted $\pi$ pulse when setting $\Omega =1$. **(c)** The second type of noise-resisted $\pi$ pulse when setting $\Delta =1$.

Then we design a five-level pulse sequence for an identity operator as

13 $$\begin{aligned} {\tilde{I}}^{(5)}&= {} U\left( {\hat{z}},\pi +\frac{\theta _{t}}{2}\right) U({\hat{z}}+\Omega _{4} {\hat{x}},\pi )U({\hat{z}}+\Omega _{3}{\hat{x}},\pi ) \nonumber \\&\quad \times U({\hat{z}}+\Omega _{2}{\hat{x}},\pi )U({\hat{z}}+\Omega _{1}{\hat{x}},\pi )U({\hat{z}}+\Omega _{0}{\hat{x}},2\pi ) \nonumber \\&\quad \times U({\hat{z}}+\Omega _{1}{\hat{x}},\pi )U({\hat{z}}+\Omega _{2}{\hat{x}},\pi )U({\hat{z}}+\Omega _{3}{\hat{x}},\pi ) \nonumber \\&\quad \times U({\hat{z}}+\Omega _{4}{\hat{x}},\pi )U\left( {\hat{z}},\pi -\frac{\theta _{t}}{2}\right) . \end{aligned}$$

Here we fix the angle parameters as $m_{0,1,2,3,4,5}=1$, $\theta _{1,2,3,4}=0$, $\theta _{5}=\frac{\theta _{t}}{2}$ and one amplitude parameter as $\Omega _{5}=0$, and utilize other amplitude quantities $\Omega _{0,1,2,3,4}$ as the free parameters.

Finally we insert the identity operator between $U({\hat{z}}+{\hat{x}},\pi )$ and $U({\hat{z}},\theta _{t})$ in Eq. (12), and we obtain a symmetric composite pulse as

14 $$\begin{aligned} U_{cp2}&= {} U({\hat{z}}+{\hat{x}},\pi )U\left( {\hat{z}},\pi +\frac{\theta _{t}}{2}\right) U( {\hat{z}}+\Omega _{4}{\hat{x}},\pi )U({\hat{z}}+\Omega _{3}{\hat{x}},\pi ) \nonumber \\&\quad \times U({\hat{z}}+\Omega _{2}{\hat{x}},\pi )U({\hat{z}}+\Omega _{1}{\hat{x}},\pi )U({\hat{z}}+\Omega _{0}{\hat{x}},2\pi ) \nonumber \\&\quad \times U({\hat{z}}+\Omega _{1}{\hat{x}},\pi )U({\hat{z}}+\Omega _{2}{\hat{x}},\pi )U({\hat{z}}+\Omega _{3}{\hat{x}},\pi ) \nonumber \\&\quad \times U({\hat{z}}+\Omega _{4}{\hat{x}},\pi )U\left( {\hat{z}},\pi +\frac{\theta _{t}}{ 2}\right) U({\hat{z}}+{\hat{x}},\pi ), \end{aligned}$$

where we denote the whole pulse as CP2. The noise insensitivity requirement gives a set of coupled equations for the variables $\Omega _{0,1,2,3,4}$. We determined the results for these adjustable parameters, which are provided in Table 2.

**Figure 3:**
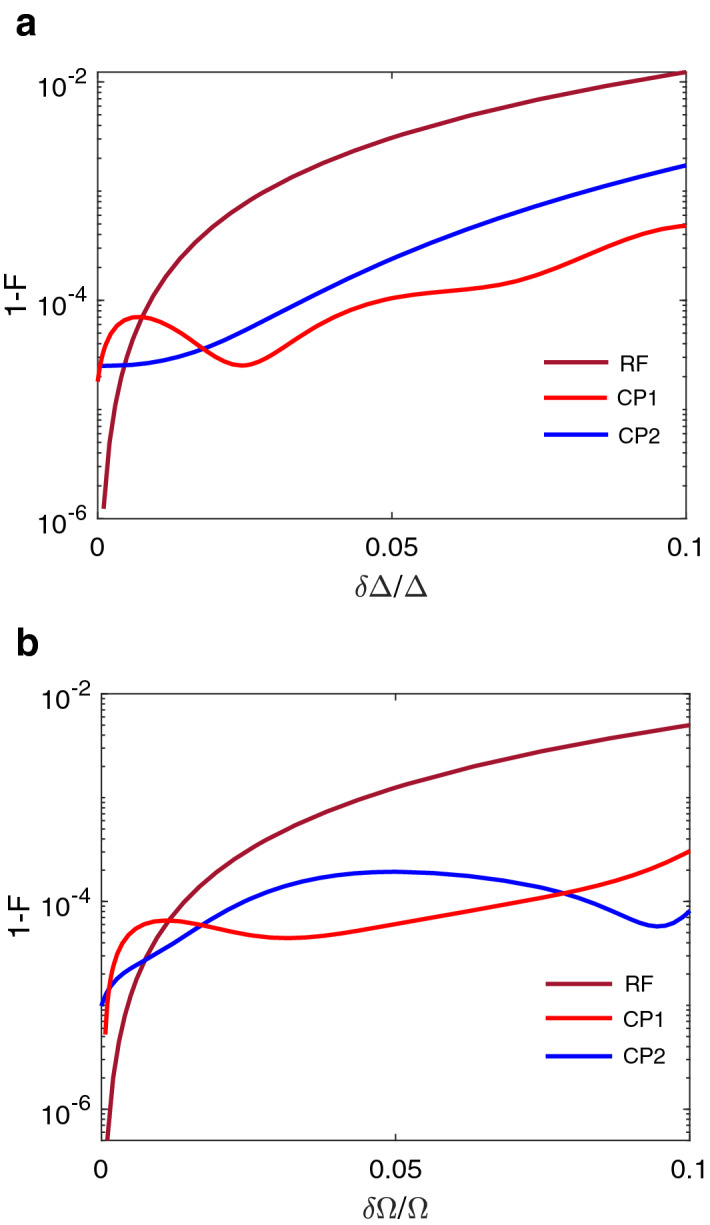
The infidelity $1-F$ of three pulses: the designed noise-protected $\pi$ pulse (denoted as CP1 when setting $\Omega =1$, and CP2 when setting $\Delta =1$, respectively), and the commonly used rectangular $\pi$ pulse (denoted as RF). **(a)** The infidelity as a function of the frequency detuning fluctuation $\delta \Delta /\Delta$. The simulation is under a noise of $\delta \Omega /\Omega =0.01$. **(b)** The infidelity as a function of the driving amplitude fluctuation $\delta \Omega /\Omega$. The evolution is under a noise of $\delta \Delta /\Delta =0.01$.

Using the parameters in the tables, we design the noise-protected $\pi$ pulses as illustrated in Fig. 2. For comparison, Fig. 2a is the commonly used rectangular pulse, and Fig. 2b,c are the composite pulses based on designed frequency detunings and driving amplitudes, respectively. We note that the rectangular pulse is a physically ideal case with approximation $\Omega \gg \Delta$. To compare the performance of different pulses, we calculate the fidelity of the pulses under the realistic noises. We note that the typical range of values of the noise-to-control-field ratio ($\frac{ \delta \Omega }{\Omega }$ or $\frac{\delta \Delta }{\Delta }$) in solid state spin system is between 0 and 0.1^17, 33, 39^. The fidelity is defined as the formula^40^

15 $$\begin{aligned} F=\text {Tr(}\sqrt{\sqrt{\rho _{0}}\rho \sqrt{\rho _{0}}}\text {)}, \end{aligned}$$

where $\rho _{0}$ is the density matrix of the system without the noises and $\rho$ is the density matrix under the noises. Figure 3 shows the infidelity $1-F$ for three $\pi$ pulses under different types of noises. In Fig. 3, we observe the noise-resisted pulses provide error compensation and the infidelity is orders of magnitude smaller than that of the common $\pi$ pulse. In Fig. 3a, we keep the amplitude noise $\frac{ \delta \Omega }{\Omega }=0.01$ and study the effect of the fluctuation of the frequency $\delta \Delta$. The CP1 $\pi$ pulse performs the best and achieves an infidelity lower than $0.01\%$ even when there is a large noise of $\frac{\delta \Delta }{\Delta }=0.1$. The CP2 $\pi$ pulse achieves the second small infidelity and the commonly used rectangular $\pi$ pulse gets the largest infidelity. In Fig. 3b, we consider the main noise which comes from the fluctuation of the amplitude $\delta \Omega$. The CP2 $\pi$ pulse performs the best with an infidelity lower than $0.001\%$ even under a large noise of $\frac{\delta \Omega }{\Omega }=0.1$. As expected, the CP1 $\pi$ pulse ranks the second and the ordinary $\pi$ pulse is the last one.

### Applications to DD pulse sequences

The DD pulse sequence consists of two basic processes^16^. One is the $\pi$ pulse around *x* axis (or other axis), which is described by the operator $U({\hat{x}},\pi )$. The other is the free evolution of the system, which is given by the operator

16 $$\begin{aligned} V({\hat{z}},\tau )=\exp \left[ -i\left( \frac{\Delta }{2}\sigma _{z}\right) \tau \right] . \end{aligned}$$

Here $\tau$ is the time interval between adjacent $\pi$ pulses. The building block of the DD sequence is written as

17 $$\begin{aligned} U_{{\hat{x}}}=V({\hat{z}},\tau )U({\hat{x}},\pi )V({\hat{z}},\tau ), \end{aligned}$$

where the subscript denotes the $\pi$ pulse around *x* axis for clarity. It is intuitive to understand the mechanism of the DD scheme: since the error in the detuning term $\Delta \tau$ has different signs before and after the $\pi$ pulse, the effect of inhomogeneous broadening or detuning fluctuations is averaged. Thus a DD pulse sequence can be constructed by repeating the basic elements.

There are kinds of DD pulse sequences, which are formed by several $\pi$ pulses in periodic or inperiodic structures^16^. Here we focus on three examples of DD sequences which are extensively applied in the field of quantum memories. The first type of DD protocol is the Carl-Purcell-Meiboom-Gilles (CPMG) sequence^41, 42^. The CPMG sequence consists of two $\pi$ pulses around *x* axis, which is the simplest extension of the conventional spin-echo. The CPMG pulse sequence can be described as

18 $$\begin{aligned} U_{CPMG}&= {} U_{{\hat{x}}}U_{{\hat{x}}} \nonumber \\&= {} V({\hat{z}},\tau )U({\hat{x}},\pi )V({\hat{z}},\tau )V({\hat{z}},\tau )U({\hat{x}} ,\pi )V({\hat{z}},\tau ). \end{aligned}$$

The second type is the so-called XY4 sequence^43^ which consists of four $\pi$ pulses with an alternation of the rotation axis around *x* and *y* directions. XY4 sequence is introduced to partially compensate amplitude errors for any initial state. Furthermore, XY4 sequence is more immune to errors in the pulses to some extent^44^. It can be written as the repetition of the building blocks:

19 $$\begin{aligned} U_{XY4}&= {} U_{{\hat{y}}}U_{{\hat{x}}}U_{{\hat{y}}}U_{{\hat{x}}} \nonumber \\&= {} V({\hat{z}},\tau )U({\hat{y}},\pi )V({\hat{z}},\tau )V({\hat{z}},\tau )U({\hat{x}} ,\pi )V({\hat{z}},\tau ) \nonumber \\&\quad \times V({\hat{z}},\tau )U({\hat{y}},\pi )V({\hat{z}},\tau )V({\hat{z}},\tau )U( {\hat{x}},\pi )V({\hat{z}},\tau ). \end{aligned}$$

The third type is the UDD sequence^25^, which has a non-uniform inter-pulse delay. The UDD sequence is introduced to minimize dephasing effect for given number of pulses. The UDD scheme can be represented as *N* successive $\pi$ pulses (indexed by *j*) at times $\tau _{j}=\tau _{D}\sin ^{2}[j\pi /(2N+2)]$, where $\tau _{D}$ is the total duration of the pulse sequence.

In the DD applications for quantum memories, to reach longer storage time, one can repeat the sequences several times, such as $U_{{\hat{x}}}U_{{\hat{x}} }...U_{{\hat{x}}}U_{{\hat{x}}}$. The final DD sequences consist of *N* successive rotations by a nominal angle $\pi$ around certain axis, where *N* denotes the numbers of $\pi$ pulses or the length of the sequence. Under ideal conditions, the evolution operator of the whole DD sequence corresponds to the identity operation. However, in realistic situations, we have to consider the the cumulative effect of pulse imperfections. If the actual rotation of each pulse differs by the fluctuations $\delta \Delta$ and $\delta \Omega$, the error accumulates over the *N* pulses and the total propagator of the DD sequence becomes

20 $$\begin{aligned} U_{dd}=I_{0}+i\sum _{k=x,y,z}D_{k}\sigma _{k}, \end{aligned}$$

where $D_{k}$ denotes the error due to the noises in each pulse.

**Figure 4:**
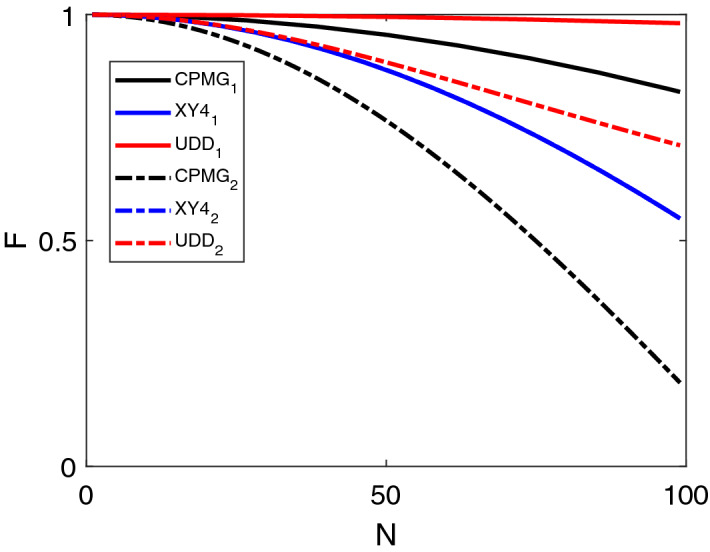
The fidelity *F* vs the number of $\pi$ pulses in the DD sequence. The subscript 1 and solid line denote the DD sequence constructed by the noise-resisted $\pi$ pulse, while subscript 2 and dotted line label the sequence using the rectangular shape $\pi$ pulse. The noises in the simulations are set as $\frac{\delta \Delta }{ \Delta }=\frac{\delta \Omega }{\Omega }=0.01$.

To compensate the errors due to the noises in the detuning and the amplitudes, we combine the noise-resisted pulses with DD sequences. We can use the above designed noise-resisted $\pi$ pulse to replace the noisy $\pi$ pulse in the DD sequences. For comparison, we also consider the DD sequence using the common rectangular shape $\pi$ pulse. To illustrate the improvement, we investigate the fidelity of different DD pulse sequences. In many cases, we want to quantify the agreement not between states, but between two evolutions. The corresponding process fidelity can be defined in close analogy to the state fidelity^40^:

21 $$\begin{aligned} F=\frac{{\vert }\text {Tr}(U_{A}^{\dagger }U_{B}){\vert }}{{\sqrt{\text {Tr}(U_{A}^{\dagger }U_{A})\text {Tr}(U_{B}^{\dagger }U_{B})}}} \end{aligned}$$

where $U_{A}$ is the target propagator, such as unity in the ideal situation, and $U_{B}$ is the actual propagator implemented by the DD pulses.

Figure 4 shows the simulated performance of the DD sequences as a function of the length of the pulse sequence. The actual propagator which is generated by the common rectangular $\pi$ pulse has vanishing overlap with the target propagator, and the fidelity of the operation is zero for long-term sequence. In contrast, we find that the fidelity of the combined DD sequence is significantly high even for the long-duration sequence, in particular for the UDD case. These results illustrate the DD sequences are more robust and effective using the noise-resisted $\pi$ pulses. As the conventional DD sequences have been performed to prolong the lifetime of a quantum memory^17, 45^, one can expect that the improved DD method proposed here can extend the lifetime of a quantum memory significantly as the number of the DD pulses increases.

Figure 4 shows that inserting the noise-protected pulse into the XY4 sequence has little improvement. We suspect there are two possible reasons. One reason is that XY4 sequence is defined as an alternation of pulses in *x* and *y* direction, thus the rotation effect around two axes would cancel each other out. The other reason is that the XY4 sequence itself is more immune to errors in the pulses to some extent^44^, thus the complex designed noise-resistant pulses would hardly provide further improvement. We note that here we focus on the number of the pulse sequences, while the process fidelity would also depend on the time interval. The present results are applicable to the case of static noise. A detailed study on the time interval requires a model including the dynamical noise effect.

## Conclusion

In conclusion, we propose the design of DD pulse sequences for robust manipulations in quantum memories based on noise-resisted pulse techniques. We construct a composite pulse to zero the errors to the first order of noises during the operations. Furthermore, we design the noise-protected $\pi$ pulses and analyze multiple $\pi$ pulses on a long time scale. Our characterization of the fidelity of the operation process shows that these pules can achieve high fidelity even under realistic noises. Similar to the composite pulses in the NMR field^46^, our approach average out unwanted evolution by cascading primitive control operations. In contrast, our approach has the advantages that corrects both amplitude and detuning noise errors while being sufficiently flexible for incorporation into arbitrary gate operations. We also note that in the NMR field, recently a number of methods have been developed to suppress the noises by topological DD method^47–49^, or by eliminating the errors in the synthesizers^50–52^.

There are two figures of merit (performance criteria) in view of the development of a quantum memory: one is the fidelity, the other is the efficiency. The study of effects of the improved DD method on efficiency would be an interesting topic in the future work. Quantum memories are important in various quantum communication applications^53, 54^, in particular the quantum secure direct communication^55, 56^. This method will have important consequences for the goal of quantum networks based on quantum memories^4–6^, as well as for the quantum architectures to store and control quantum states using microwave quantum memories^57, 58^.

## Supplementary information

Supplementary Information.
