# Supplementary material for: A noise-resisted scheme of dynamical decoupling pulses for quantum memories
Source: Sci Rep. 2020 Sep 15;10:15089. doi: 10.1038/s41598-020-72071-x (PMC7494898; doi:10.1038/s41598-020-72071-x)
Supplement: Supplementary file 1 — Supplementary Information. [file 41598_2020_72071_MOESM1_ESM.pdf]

Supplementary Information for
A noise-resisted scheme of dynamical decoupling pulses for quantum memories

Bo Gong, Tao Tu, Xing-Yu Zhu, Ao-Lin Guo, Guang-Can Guo, and Chuan-Feng Li

Section S1: Error terms in the elementary operation

We consider the elementary operator $U(\Delta\hat{z}+\Omega\hat{x},\theta)$:

$$U(\Delta\hat{z}+\Omega\hat{x},\theta)=\exp[-i(\frac{\Delta+\delta\Delta}{2}\sigma_z+\frac{\Omega+\delta\Omega}{2}\sigma_x)\frac{\theta}{\sqrt{\Omega^2+\Delta^2}}], \tag{S1}$$

which contains the noises terms $\delta\Delta$ and $\delta\Omega$ in the exponential form. Using the general formula $e^{A+B}=e^Ae^Be^{-\frac{1}{2}[A,B]}$ for the two operators A and B, we can expand the operator $U(\Delta\hat{z}+\Omega\hat{x},\theta)$ as a series of $\delta\Delta$ and $\delta\Omega$ and obtain the expression:

$$U(\Delta\hat{z}+\Omega\hat{x},\theta)=\exp[-i(\frac{\Delta}{2}\sigma_z+\frac{\Omega}{2}\sigma_x)\frac{\theta}{\sqrt{\Omega^2+\Delta^2}}]\times(I_0-i\sum_{k=x,y,z}\epsilon_k\sigma_k). \tag{S2}$$

To the first order of $\delta\Delta$ and $\delta\Omega$, the error terms in the three directions can be explicitly given as a function of $\delta\Delta$ and $\delta\Omega$:

$$\begin{aligned}\varepsilon_x&=\frac{\Omega\Delta(\theta-\sin\theta)}{2J^3}\delta\Delta+\frac{\Omega^2\theta+\Delta^2\sin\theta}{2J^3}\delta\Omega,\\ \varepsilon_y&=\frac{\Omega(1-\cos\theta)}{2J^2}\delta\Delta+\frac{\Delta(\cos\theta-1)}{2J^2}\delta\Omega,\\ \varepsilon_z&=\frac{\Delta^2\theta+\Omega^2\sin\theta}{2J^3}\delta\Delta+\frac{\Omega\Delta(\theta-\sin\theta)}{2J^3}\delta\Omega.\end{aligned} \tag{S3}$$

Here we define $J=\sqrt{\Omega^2+\Delta^2}$ for convenience.

Section S2: Error terms in the noisy identity operator

The key step of our scheme is the construction of the noisy identity operator as

$$\tilde{I}^{(n)}=U(\Delta_n\hat{z}+\Omega_n\hat{x},m_n\pi+\theta_n)\times\tilde{I}^{(n-1)}\times U(\Delta_n\hat{z}+\Omega_n\hat{x},m_n\pi-\theta_n), \tag{S4}$$

which also can be expanded as

$$\tilde{I}^{(n)}=I_0-i\sum_{k=x,y,z}\Theta_k^{(n)}\sigma_k. \tag{S5}$$

Since this construction of the operator is a recursive form, we can apply a matrix multiplication to obtain the corresponding recursive form of the error terms [1]:

$$\begin{aligned}\Theta_x^{(n)}&=\frac{m_n\pi\Omega_n\Delta_n}{J_n^3}\delta\Delta+\frac{m_n\pi\Omega_n^2}{J_n^3}\delta\Omega+\frac{\Omega_n^2+(-1)^{m_n}\Delta_n^2\cos\theta_n}{J_n^2}\Theta_x^{(n-1)}\\ &+\frac{(-1)^{m_n}\Delta_n\sin\theta_n}{J_n}\Theta_y^{(n-1)}+\frac{\Omega_n\Delta_n\left(1-(-1)^{m_n}\cos\theta_n\right)}{J_n^2}\Theta_z^{(n-1)},\\ \Theta_y^{(n)}&=-\frac{(-1)^{m_n}\Delta_n\sin\theta_n}{J_n}\Theta_x^{(n-1)}+(-1)^{m_n}\cos\theta_n\Theta_y^{(n-1)}+\frac{(-1)^{m_n}\Omega_n\sin\theta_n}{J_n}\Theta_z^{(n-1)},\\ \Theta_z^{(n)}&=\frac{m_n\pi\Delta_n^2}{J_n^3}\delta\Delta+\frac{m_n\pi\Delta_n\Omega_n}{J_n^3}\delta\Omega+\frac{\Omega_n\Delta_n\left(1-(-1)^{m_n}\cos\theta_n\right)}{J_n^2}\Theta_x^{(n-1)}\\ &-\frac{(-1)^{m_n}\Omega_n\sin\theta_n}{J_n}\Theta_y^{(n-1)}+\frac{\Delta_n^2+(-1)^{m_n}\Omega_n^2\cos\theta_n}{J_n^2}\Theta_z^{(n-1)}.\end{aligned} \tag{S6}$$

Here we define $J_n=\sqrt{\Omega_n^2+\Delta_n^2}$ for clarity. Therefore, we have the relations between level $n-1$ and n, and can obtain the analytic expression of the error terms $\Theta_k^{(n)}$ for any level n.

Section S3: Determining the composite pulse parameters

Taking into account both the noisy target operator and the noisy identity operator, we obtain the total equation for the error terms of the composite pulse:

$$U_c=U_0(\Delta_t\hat{z}+\Omega_t\hat{x},\theta_t)(I_0-i\sum_{k=x,y,z}\Gamma_k\sigma_k), \tag{S7}$$

with $\Gamma_k = \varepsilon_k + \Theta_k^{(n)}$. Our goal is that the composite pulse is immune to the leading order of noises, namely,

$$\varepsilon_k + \Theta_k^{(n)} = 0. \tag{S8}$$

Using Eq. (S3) and (S6) which define the error terms ε_k and $\Theta_k^{(n)}$, we can rewrite Eq. (S8) as a series of coupled equations for the variables Ω_n and Δ_n. These coupled equations are highly non-linear and are solved using the numerical algorithm. If one has the desired solution (namely, all parameters are non-negative real numbers), then the procedure is finished. Otherwise, one would try to alter the searching ranges until finding a satisfactory solution. We numerically solved these coupled equations to give the pulse parameters Ω_n and Δ_n, where two examples are shown in Table 1 and 2 in the main text.

[1] Wang, X., et al. Composite pulses for robust universal control of singlet–triplet qubits *Nat. Commun.* **3**, 997 (2012).
